# Association of basal thyroid function with clinical outcomes in patients with recurrent or metastatic nasopharyngeal carcinoma treated with PD-L1 inhibitor KL-A167: a multicenter post hoc analysis

**DOI:** 10.1530/EC-26-0083

**Published:** 2026-04-28

**Authors:** Keliang Chen, Haohan Fan, Shihong Xu, Jiacheng Li, Junyou Ge, Yan Qing, Youneng Wei, Yuping Xie, Xingchen Peng

**Affiliations:** ^1^ Department of Biotherapy, Cancer Center, West China Hospital, Sichuan University, Chengdu, Sichuan, PR China; ^2^ College of Medical and Life Sciences, Chengdu University of Traditional Chinese Medicine, Chengdu, Sichuan, China; ^3^ Sichuan Kelun-Biotech Biopharmaceutical Co., Ltd, Chengdu, Sichuan, China; ^4^ Nuclear Physics and Medical Research Key Laboratory of Sichuan Province, Sichuan University, Chengdu, Sichuan, PR China

**Keywords:** nasopharyngeal carcinoma, PD-L1 inhibitors, thyroid-stimulating hormone, biomarkers, prognosis

## Abstract

**Objective:**

While thyroid dysfunction during PD-L1 inhibitor therapy correlates with efficacy in recurrent/metastatic nasopharyngeal carcinoma, the prognostic value of basal thyroid function remains unclear. This study investigated the relationship between baseline serum thyroid-stimulating hormone (TSH) and clinical outcomes.

**Methods:**

We conducted a multicenter, retrospective analysis of 153 recurrent/metastatic nasopharyngeal carcinoma (R/M NPC) patients from a prospective phase 2 trial of the PD-L1 inhibitor KL-A167. Patients were stratified by baseline TSH levels. Multivariate Cox and logistic regression models were used to analyze progression-free survival (PFS), overall survival (OS), and objective response rate (ORR).

**Results:**

High basal TSH (*n* = 58) was independently associated with significantly prolonged OS (HR 0.56, 95% CI: 0.36–0.88; *P* = 0.011) and PFS (HR 0.60, 95% CI: 0.41–0.87; *P* = 0.008) compared to the low/normal TSH group (*n* = 95). Although ORR was numerically higher in the high TSH group (27.6 vs 17.9%), the difference was not statistically significant (*P* = 0.23). Subgroup analyses indicated consistent benefits across most clinical strata. Thyroid immune-related adverse events occurred in 32/153 (20.9%), similarly between groups (20.7 vs 21.1%, *P* = 0.957), and were not significantly associated with either OS (HR 0.65, 95% CI: 0.38–1.11, *P* = 0.117) or PFS (HR 0.88, 95% CI: 0.54–1.44, *P* = 0.613) by time-varying Cox regression.

**Conclusion:**

Elevated basal TSH levels are independently associated with improved survival in R/M NPC patients receiving KL-A167. Baseline TSH may serve as a simple, noninvasive biomarker for risk stratification and personalizing immunotherapy in this population.

## Introduction

Nasopharyngeal carcinoma (NPC) is a highly aggressive malignancy distinguished by a unique geographical distribution, being notably endemic to North Africa, Southeast Asia, and particularly Southern China (1, 2, 3, 4). Clinically, the disease is characterized by high mortality, with 15% of patients presenting with distant metastases at the time of initial diagnosis (5).

Over the past few decades, despite continuous advancements in therapeutic strategies (6, 7), the rates of locoregional recurrence and distant metastasis remain persistently high, with retrospective analyses indicating locoregional recurrence rates of 5–15% and distant metastasis rates of 15–30% even in the modern era (8, 9, 10, 11). Consequently, outcomes for patients with recurrent or metastatic (R/M) NPC are often heterogeneous and suboptimal.

In recent years, immune checkpoint inhibitors (ICIs), particularly programmed cell death-ligand 1 (PD-L1) inhibitors, have revolutionized the treatment landscape for R/M NPC (6, 12, 13). KL-A167, a novel antibody targeting PD-L1, has demonstrated a favorable efficacy and safety profile in patients with R/M NPC who are refractory to standard first-line regimens (14). Despite significant advancements, the clinical response to ICI therapy remains heterogeneous, with only a subset of patients achieving durable tumor regression (15, 16, 17). Identifying prognostic biomarkers that can stratify patients according to their likelihood of response and survival outcomes is, therefore, of paramount importance (1, 13, 18, 19).

Thyroid function has emerged as a potentially important indicator of ICI efficacy in various cancer types (20). Recent studies have suggested that thyroid dysfunction, particularly thyroiditis and thyroid hormone abnormalities, occurring during ICI therapy may be associated with improved clinical outcomes (21, 22). However, the relationship between basal thyroid function and ICI response in NPC remains poorly understood.

The thyroid is a critical regulator of metabolic processes and immune system function. The PD-1/PD-L1 pathway, which is targeted by ICIs, is expressed in thyroid tissue, making it particularly susceptible to immune-related adverse events (irAEs) (23, 24). Indeed, thyroid dysfunction is one of the most common irAEs observed in patients receiving ICI therapy (25, 26, 27, 28, 29). This phenomenon may reflect a robust immune response not only against tumor cells but also against normal thyroid tissue.

The current study aims to investigate the relationship between basal thyroid function parameters, particularly TSH levels, and the clinical response to KL-A167 in patients with recurrent or metastatic NPC. We hypothesize that higher basal TSH levels may be associated with improved treatment response and longer survival outcomes. Our findings may provide important insights into the mechanisms underlying KL-A167 efficacy and offer a simple, noninvasive method for predicting treatment outcomes in this patient population.

This is a multicenter, retrospective study involving 153 patients with R/M NPC treated with KL-A167. Basal thyroid function parameters were collected before the initiation of KL-A167 therapy, and their association with objective response rate (ORR), disease control rate (DCR), progression-free survival (PFS), and overall survival (OS).

To the best of our knowledge, this is the first study specifically examining the relationship between basal thyroid function and response to KL-A167 therapy in R/M NPC patients. Our results may have clinical relevance by providing additional information for treatment assessment and patient stratification in KL-A167 therapy.

## Methods

### Study design and participants

This retrospective investigation utilized data from a multicenter, prospective phase 2 trial carried out across 42 medical institutions in China. The cohort comprised 153 subjects with histologically verified R/M NPC who underwent treatment with KL-A167, a humanized anti-PD-L1 monoclonal antibody. This study is registered with ClinicalTrials.gov. All study procedures complied with the ethical standards outlined in the Declaration of Helsinki and the good clinical practice (GCP) guidelines issued by the International Council for Harmonisation of Technical Requirements for Pharmaceuticals for Human Use (ICH). Detailed eligibility requirements, exclusion criteria, and the study enrollment flow chart are presented in the Supplementary Appendix (Supplementary Fig. 1 (see section on Supplementary materials given at the end of the article)).

### Clinical endpoints

The primary efficacy assessments included the ORR, DCR, PFS, and OS. An independent review committee (IRC) evaluated tumor responses based on RECIST v1.1 guidelines. ORR was defined as the percentage of participants achieving a confirmed complete response (CR) or partial response (PR). DCR encompassed the total proportion of patients exhibiting CR, PR, or stable disease (SD). PFS was calculated as the interval from the initial administration of KL-A167 to documented disease progression or death. OS was measured from the date of treatment initiation until death from any cause.

### Statistical analysis

Participants were stratified into high and low/normal TSH cohorts based on their baseline thyroid-stimulating hormone levels. Baseline TSH levels were obtained from routine peripheral blood tests conducted within a two-week window prior to the initiation of KL-A167 therapy. Classification relied on the specific reference ranges of each participating center, which may have introduced minor variations in cutoff thresholds. Patients were categorized relative to their local upper limit of normal (ULN). The cohort was divided into a ‘high TSH’ group (TSH strictly > local ULN) and a ‘low/normal TSH’ group (TSH ≤ local ULN). We employed Cox proportional hazards models to evaluate associations between TSH status and survival outcomes (PFS and OS). Survival probabilities were visualized using Kaplan–Meier curves and compared between groups.

To verify the prognostic value of TSH in the context of PD-L1 blockade, we developed multifactorial models adjusting for clinically relevant factors. We further explored potential effect modifiers, including age, sex, body mass index (BMI), ECOG performance status, TNM staging (T and N stages), number of chemotherapy cycles, and surgical history. Multiplicative interactions were assessed via likelihood ratio tests, while additive interactions were evaluated by calculating the relative excess risk due to interaction. Given the retrospective nature of the study, a post hoc minimum detectable effect size (MDE) analysis was performed to contextualize the statistical power available for each endpoint. For the binary endpoint (ORR), the MDE was calculated using a two-sided two-proportion z-test based on the fixed sample sizes (*n*_1_ = 58 for high TSH; *n*_0_ = 95 for low/normal TSH), a two-sided alpha of 0.05, and a target power of 80%. For survival endpoints (OS and PFS), the MDE was expressed as the minimum hazard ratio (HR) detectable at 80% power, derived from the observed number of events (102 for OS; 139 for PFS), the allocation ratio between groups, a two-sided alpha of 0.05, and 80% power. Data processing was executed using R software (version 4.3.2; R Foundation for Statistical Computing, Austria). Statistical significance was defined as a two-sided *P*-value of less than 0.05.

## Results

### Baseline characteristics

A total of 153 patients with recurrent or metastatic NPC who received KL-A167 were included in this retrospective analysis (Table 1). Based on baseline serum TSH levels, the study population was stratified into two cohorts: the low/normal TSH group, comprising 95 patients (62.1%), and the high TSH group, comprising 58 patients (37.9%). During KL-A167 therapy, ICI-related thyroiditis (encompassing both hypothyroidism and hyperthyroidism) was observed in 32 patients (20.9%). Notably, the incidence of thyroiditis was evenly distributed across the baseline TSH groups, occurring in 20.7% (12/58) of patients in the high TSH group and 21.1% (20/95) of patients in the low/normal TSH group (*P* = 0.957). The demographic and clinicopathological characteristics were well balanced between the two groups, with no statistically significant differences observed.

**Table 1:** Comparisons of the study population with different TSH status at baseline.

	TSH low/normal *n*(%)	TSH high *n*(%)	*P* value
*n* (%)	95 (100)	58 (100)	
Age (years, mean (SD))	48.34 (10.21)	46.29 (9.06)	0.212
Gender (%)			0.214
Male	81 (85.3)	44 (75.9)	
Female	14 (14.7)	14 (24.1)	
BMI (%)			0.663
Low (≤18.5)	20 (21.1)	9 (15.5)	
Normal (18.5–24)	57 (60.0)	36 (62.1)	
High (≥24)	18 (18.9)	13 (22.4)	
ECOG PS (%)			0.465
0	34 (35.8)	25 (43.1)	
1	61 (64.2)	33 (56.9)	
T stage (%)			0.325
T0	19 (20.0)	17 (29.3)	
T1	4 (4.2)	2 (3.4)	
T2	5 (5.3)	5 (8.6)	
T3	15 (15.8)	7 (12.1)	
T4	22 (23.2)	6 (10.3)	
Tx	30 (31.6)	21 (36.2)	
*n* stage (%)			0.291
*n*0	28 (29.5)	20 (34.5)	
*n*1	4 (4.2)	6 (10.3)	
*n*2	20 (21.1)	6 (10.3)	
*n*3	17 (17.9)	9 (15.5)	
Nx	26 (27.4)	17 (29.3)	
Past surgical history (%)			0.366
Yes	28 (29.5)	22 (37.9)	
No	67 (70.5)	36 (62.1)	
Number of chemotherapies (mean (SD))	6.46 (2.76)	6.88 (3.26)	0.4

Abbreviations: ECOG PS, Eastern Cooperative Oncology Group performance status; BMI, body mass index (calculated as weight in kilograms divided by height in meters squared).

### Tumor response

**Figure 1:**
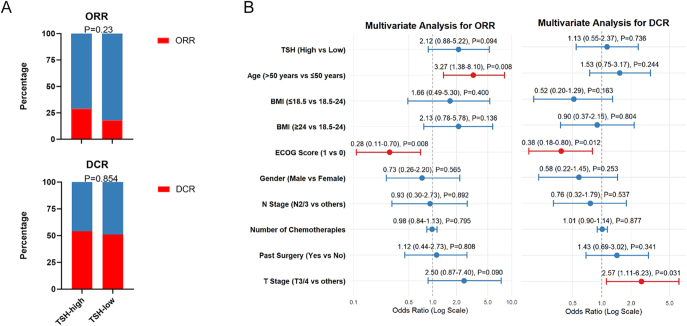
Tumor response and multivariable logistic regression analysis. (A) ORR and DCR. (B) Multivariable logistic regression analysis for ORR and DCR.

Tumor response was evaluated in all eligible patients according to RECIST v1.1 criteria. In line with the observed survival trends, the ORR was numerically higher in the high TSH group, although this difference did not reach statistical significance (*P* = 0.23). The ORR was numerically higher in the high TSH cohort, reaching 27.6% (95% CI: 0.167–0.409, *n* = 16) compared to 17.9% (95% CI: 0.108–0.271, *n* = 17) in the low/normal TSH cohort (*P* = 0.23) (Fig. 1A).

Furthermore, the DCR, comprising patients with complete response, partial response, or stable disease, showed no significant difference between the high and low/normal TSH groups. The DCR was 53.4% (95% CI: 0.399–0.667, *n* = 31) for patients with high basal TSH levels versus 50.5% (95% CI:0.401–0.609, *n* = 48) for those with low/normal basal levels (*P* = 0.854) (Fig. 1A).

To determine whether the association between basal TSH levels and tumor response was influenced by other baseline characteristics, multivariable logistic regression analyses were performed for both ORR and DCR, as illustrated in Fig. 1B. After adjustment for relevant covariates, including age, BMI, gender, ECOG performance status, and disease stage, high basal TSH levels showed a trend toward association with improved therapeutic response, although the associations did not reach statistical significance. Specifically, for ORR, the multivariable model yielded an adjusted odds ratio (OR) of 2.12 (95% CI: 0.88–5.22; *P* = 0.094).

### Survival analysis

**Figure 2:**
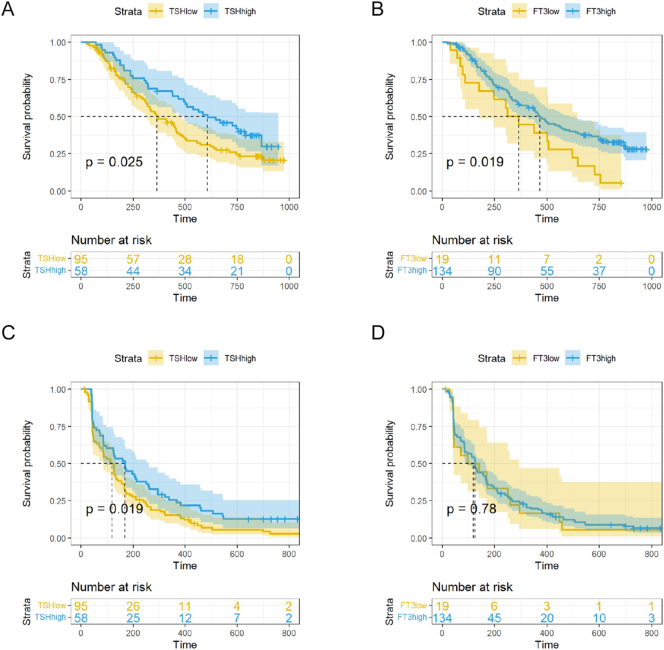
Kaplan–Meier estimates of OS and PFS. (A) OS according to baseline TSH status. (B) OS according to baseline FT3 status. (C) PFS according to baseline TSH status. (D) PFS according to baseline FT3 status.

The survival outcomes, assessed via Kaplan–Meier analysis, demonstrated a distinct prognostic advantage for patients with higher basal TSH levels. In the analysis of overall survival (OS), the high TSH cohort achieved significantly superior survival rates relative to the low/normal TSH cohort (Fig. 2A). Consistently, a similar trend was observed for PFS, where patients in the high TSH group exhibited a statistically significant prolongation in the time to disease progression or death compared to those in the low/normal TSH group (Fig. 2C). This separation of the survival curves highlights the potential of basal TSH as a prognostic biomarker in KL-A167-treated patients. These findings suggest that elevated basal TSH levels are associated with more favorable long-term outcomes in patients treated with KL-A167. A similar trend in OS was observed among patients stratified by baseline FT3 levels (Fig. 2B); however, no statistically significant difference was detected in PFS between the groups (Fig. 2D).

### Univariate Cox regression analysis

**Figure 3:**
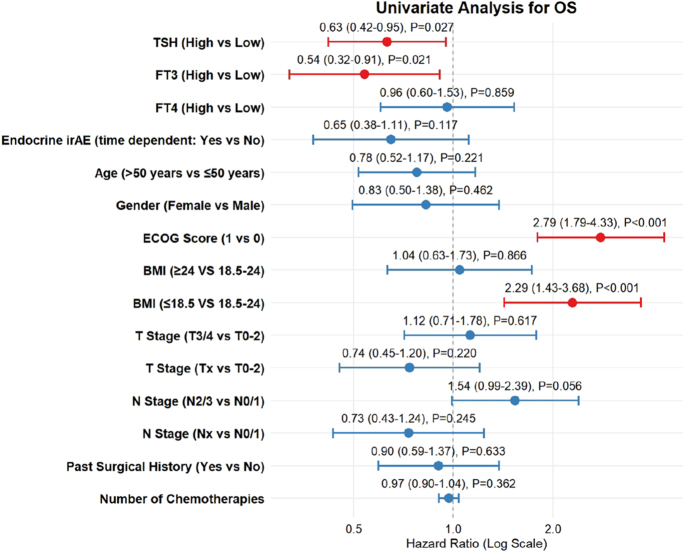
Univariate Cox regression analysis for OS.

**Figure 4:**
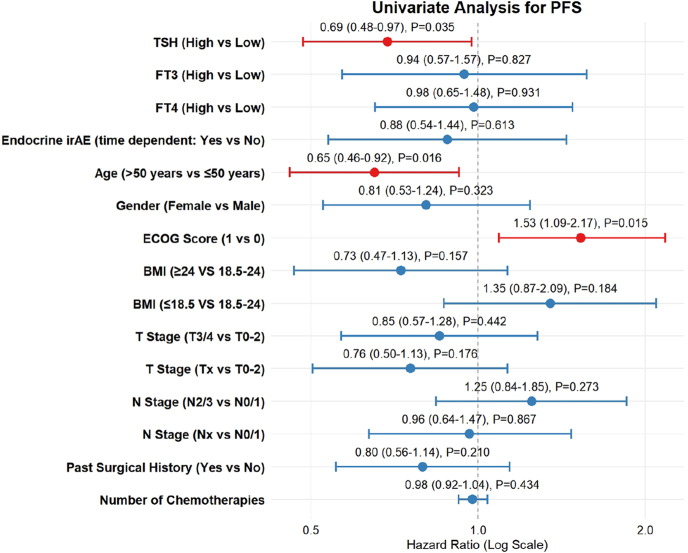
Univariate Cox regression analysis for PFS.

Univariate Cox proportional hazards regression was conducted to quantify the impact of basal TSH levels and other potential prognostic factors on survival outcomes. For OS (Fig. 3), high basal TSH levels were significantly associated with a reduced risk of mortality. The calculated HR was 0.63 (95% CI: 0.42–0.95), indicating a 37% improvement in overall survival probability for patients with high TSH levels (*P* = 0.027). Similarly, for PFS (Fig. 4), the analysis identified high basal TSH as a significant protective factor. The HR for disease progression or death in the high TSH group was 0.66 (95% CI: 0.47–0.94), corresponding to a 34% reduction in the risk of progression compared to the now/normal TSH group (*P* = 0.021). These univariate results underscore the strong association between basal thyroid function and clinical efficacy. In addition, a univariable Cox regression analysis, in which thyroid irAE was treated as a time-varying covariate, showed that thyroid irAE occurrence was not significantly associated with either OS (HR 0.65, 95% CI: 0.38–1.11, *P* = 0.117) or PFS (HR 0.88, 95% CI: 0.54–1.44, *P* = 0.613).

### Multivariate Cox regression analysis

To determine whether the prognostic value of basal TSH was independent of potential confounders, a multivariate Cox regression analysis was performed, adjusting for clinically relevant factors, including age, gender, ECOG performance status, BMI, and tumor staging (T and N stages). The multivariate model demonstrated that high basal TSH status remained significantly associated with both OS and PFS.

**Figure 5:**
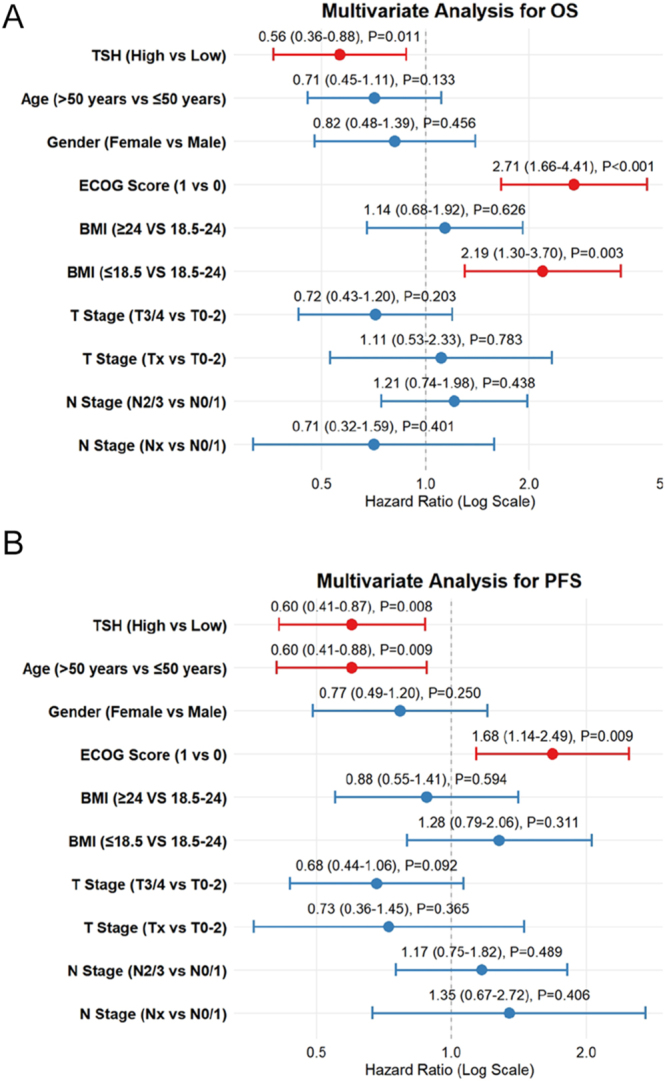
Multivariate Cox proportional hazards models for OS (A) and PFS (B).

Specifically, for OS, patients with high TSH levels exhibited a significantly reduced risk of death (HR 0.56; 95% CI: 0.36–0.88; *P* = 0.011). In this model, higher ECOG score (HR 2.71; 95% CI: 1.66–4.41; *P* < 0.001) and low BMI (HR 2.19; 95% CI: 1.30–3.70; *P* = 0.003) were associated with poorer survival outcomes. Regarding PFS, high basal TSH was similarly associated with a favorable outcome (HR 0.60; 95% CI: 0.41–0.87; *P* = 0.008), independent of age and ECOG score, which were also showed significant associations in the analysis (Fig. 5).

These results indicate that the survival benefit associated with high TSH is not merely a reflection of favorable baseline characteristics but is associated with better survival outcomes in the context of KL-A167 therapy.

### Subgroup analysis

Exploratory subgroup analyses were performed to evaluate the consistency of the survival benefit across various clinical strata. Regarding PFS, the protective effect of high TSH was evident across most subgroups (Table 2). Notably, male patients derived a significant PFS benefit (HR 0.58, 95% CI: 0.38–0.87; *P* = 0.009), as did patients aged ≤50 years (HR 0.58, 95% CI: 0.35–0.96; *P* = 0.03). The benefit was also pronounced in patients with a higher tumor burden, specifically those with N2–N3 nodal disease (HR 0.44, 95% CI: 0.21–0.92; *P* = 0.03) and T3–T4 tumor stage (HR 0.44, 95% CI: 0.20–0.97; *P* = 0.04). In addition, patients with an ECOG performance status of 1 (HR 0.53, 95% CI: 0.32–0.88; *P* = 0.01) and those with no history of surgery (HR 0.53, 95% CI: 0.33–0.85; *P* = 0.008) showed significant improvements. Interestingly, patients with a BMI ≥ 24 exhibited a substantial reduction in progression risk (HR 0.35, 95% CI: 0.13–0.97; *P* = 0.04). Interaction tests revealed no significant heterogeneity across these subgroups, suggesting a consistent therapeutic effect.

**Table 2:** Subgroup analysis for PFS.

Subgroup	TSH low/normal	TSH high	HR (95%CI)	*P* value	*P* value for interaction
All people	95	58	0.61 (0.43, 0.89)	0.009	
Age					0.9
≤50 years	54	41	0.58 (0.35, 0.96)	0.03	
>50 years	41	17	0.51 (0.24, 1.05)	0.07	
Gender					0.41
Male	81	44	0.58 (0.38, 0.87)	0.009	
Female	14	14	0.68 (0.27, 1.67)	0.4	
ECOG PS					0.3
0	34	25	0.73 (0.39, 1.33)	0.3	
1	61	33	0.53 (0.32, 0.88)	0.01	
BMI					0.94
≤18.5	20	9	0.40 (0.11, 1.37)	0.14	
18.5–24	57	36	0.62 (0.38, 0.99)	0.05	
≥24	18	13	0.35 (0.13, 0.97)	0.04	
Node stage					0.54
Nx and *n*0–*n*1	58	43	0.71 (0.46, 1.12)	0.12	
*n*2–*n*3	37	15	0.44 (0.21, 0.92)	0.03	
Tumor stage					0.92
Tx and T0–T2	58	45	0.67 (0.44, 1.03)	0.07	
T3–T4	37	13	0.44 (0.20, 0.97)	0.04	
Past surgical history					0.92
No	67	36	0.53 (0.33, 0.85)	0.008	
Yes	28	22	0.72 (0.35, 1.47)	0.37	

Abbreviations: ECOG PS, Eastern Cooperative Oncology Group performance status; BMI, body mass index (calculated as weight in kilograms divided by height in meters squared).

For OS, the subgroup analysis yielded comparable findings (Table 3). Significant survival advantages were observed in male patients (HR 0.54, 95% CI: 0.33–0.88; *P* = 0.01) and younger patients (≤50 years; HR 0.54, 95% CI: 0.31–0.94; *P* = 0.03). A particularly strong association was noted in the high BMI subgroup (≥24), where the hazard ratio for death was 0.27 (95% CI: 0.09–0.83; *P* = 0.02), with a borderline significant interaction *P* value of 0.06, suggesting that higher body mass might amplify the protective effect of TSH. Conversely, while trends favored the high TSH group in other subsets, such as those with earlier tumor stages or female gender, these did not reach statistical significance, likely due to smaller sample sizes in these strata.

**Table 3:** Subgroup analysis for OS.

Subgroup	TSH low/normal	TSH high	HR (95%CI)	*P* value	*P* value for interaction
All people	95	58	0.62 (0.40, 0.96)	0.03	
Age					0.64
≤50 years	54	41	0.54 (0.31, 0.94)	0.03	
>50 years	41	17	0.76 (0.33, 1.75)	0.51	
Gender					0.13
Male	81	44	0.54 (0.33, 0.88)	0.01	
Female	14	14	0.93 (0.30, 2.88)	0.9	
ECOG PS					0.7
0	34	25	0.59 (0.26, 1.34)	0.2	
1	61	33	0.65 (0.38, 1.11)	0.1	
BMI					0.06
≤18.5	20	9	0.67 (0.19, 2.28)	0.52	
18.5–24	57	36	0.65 (0.35, 1.21)	0.17	
≥24	18	13	0.27 (0.09, 0.83)	0.02	
Node stage					0.88
Nx and *n*0–*n*1	58	43	0.71 (0.39, 1.29)	0.3	
*n*2∼*n*3	37	15	0.58 (0.28, 1.19)	0.1	
Tumor stage					
Tx and T0–T2	58	45	0.69 (0.40, 1.16)	0.16	0.97
T3–T4	37	13	0.46 (0.20, 1.05)	0.06	
Past surgical history					0.46
No	67	36	0.67 (0.39, 1.13)	0.1	
Yes	28	22	0.53 (0.20, 1.39)	0.2	

Abbreviations: ECOG PS, Eastern Cooperative Oncology Group performance status; BMI, body mass index (calculated as weight in kilograms divided by height in meters squared).

### Detectable effect size analysis

Although the ORR was numerically higher in the high TSH group (27.6 vs 17.9%, *P* = 0.23), MDE analysis indicated that 80% statistical power required a minimum ORR of 40.1% in the high TSH group – corresponding to a minimum absolute difference of 22.2 percentage points. Because the observed absolute difference was only 9.7%, the study was substantially underpowered for this endpoint. The nonsignificant ORR result should, therefore, not be interpreted as evidence of no effect, but rather reflects an insufficient sample size to detect a difference of this magnitude.

For survival endpoints, the MDE analysis indicated that – given 102 OS events and 139 PFS events – the study had 80% power to detect an HR of ≤0.56 for OS and ≤0.61 for PFS. The observed univariate HRs of 0.63 for OS (*P* = 0.027) and 0.66 for PFS (*P* = 0.021) were modestly above these thresholds, indicating that the study operated at below the prespecified 80% power level for the effect sizes actually observed. Despite this, both endpoints achieved statistical significance. These findings should be interpreted with appropriate caution: the associations are unlikely to be attributable to chance alone, but the confidence intervals remain relatively wide (OS 95% CI: 0.42–0.95; PFS 95% CI: 0.47–0.94), and this study may be underpowered to precisely estimate the true effect magnitude. Prospective validation in larger cohorts is, therefore, warranted to confirm these survival benefits.

## Discussion

This study demonstrates a significant association between higher basal TSH levels and improved PFS and OS in patients with recurrent/metastatic NPC receiving KL-A167. This finding is consistent with previous reports in other cancer types, where thyroid dysfunction occurring during ICI treatment has been associated with better clinical outcomes (20). However, our study extends these observations by highlighting the potential role of basal thyroid function as a prognostic biomarker in KL-A167-treated patients.

One plausible mechanism underlying this association is the concept of ‘immunological cross-reactivity’. Thyroid tissue possesses the potential for high PD-L1 expression, particularly under inflammatory conditions, serving as a protective mechanism that is disrupted by ICIs (28). It is hypothesized that patients with higher basal TSH levels may have a preexisting state of thyroid inflammation or altered immune surveillance, which could be amplified by KL-A167 therapy. This increased immune activity against thyroid tissue might correlate with a more robust antitumor response, as both processes involve the activation of the immune system.

Another possible explanation is the role of thyroid hormones in regulating tumor metabolism and immune evasion mechanisms. Thyroid hormones influence cellular metabolism and can modulate the expression of various genes involved in immune cell function (30). Patients with higher TSH levels often have subclinical hypothyroidism, which may alter tumor metabolism in a way that makes them more vulnerable to immune checkpoint inhibitors. Specifically, hypothyroidism is associated with decreased metabolic activity, which could reduce the ability of tumor cells to evade immune detection (31, 32).

In addition, the borderline interaction (*P* = 0.06), suggesting that BMI amplifies the protective effect of TSH, must be interpreted cautiously. Given the well-established physiological positive correlation between BMI and TSH levels, this observed amplification may be confounded by intrinsic collinearity. Rather than a true synergistic effect, TSH in this context might partially serve as a proxy for adiposity-related metabolic alterations. Future studies are needed to disentangle this physiological axis.

Moreover, the thyroid itself is a well-recognized, highly immunogenic organ, and the onset of immune-related thyroiditis during ICI treatment has been widely established as a surrogate marker of robust systemic immune activation. Previous studies have consistently demonstrated that the occurrence of thyroid-related irAEs is closely correlated with favorable treatment response and survival outcomes in multiple tumor types treated with ICIs (21, 33, 34, 35). However, in our analysis, we found no statistically significant association between basal thyroid function and the incidence of treatment-related immune thyroiditis (*P* = 0.957). This key finding indicates that the prognostic survival benefit conferred by elevated basal TSH is independent of the development of thyroid irAEs during KL-A167 therapy, rather than being mediated by promoting the onset of treatment-related thyroid inflammatory events. This observation further extends prior research: unlike treatment-induced thyroid dysfunction which can only be identified retrospectively after treatment initiation, basal TSH levels serve as an easily accessible, pretreatment prognostic biomarker in KL-A167-treated patients that enables upfront risk stratification and response prediction, without relying on the occurrence of on-treatment adverse events. These findings offer substantial insights into the clinical management of recurrent or metastatic NPC, establishing basal TSH as a valuable, noninvasive biomarker for pretreatment risk stratification. By predicting treatment response and survival outcomes prior to the initiation of KL-A167, basal TSH levels may help inform therapeutic planning and patient stratification.

Furthermore, this study builds upon existing evidence that identifies treatment-induced thyroid dysfunction as a surrogate marker for therapeutic efficacy by demonstrating that the baseline thyroid status independently possesses significant prognostic value (21). Crucially, the association between elevated basal TSH and improved survival addresses the debate regarding preexisting thyroid abnormalities. Rather than serving as a contraindication, subclinical hypothyroidism may mark a subset of patients poised to derive significant benefit from KL-A167, suggesting that therapy should not be withheld based solely on this condition. Ultimately, integrating basal TSH with established markers, such as PD-L1 expression and clinical covariates like ECOG performance status, could facilitate the development of robust, multidimensional nomograms to optimize precision medicine in NPC.

The interpretation of our findings requires caution due to several inherent limitations. As a retrospective analysis, this study is subject to potential selection bias and data heterogeneity, while the relatively modest cohort size (*n* = 153) may constrain the broader generalizability of the results. Crucially, this generalizability is further limited because our cohort consisted exclusively of Chinese patients treated with KL-A167, a drug currently approved and utilized only in China. Therefore, these results may not be directly extrapolatable to other ethnic populations or global clinical practices. In addition, our analysis did not stratify patients by NPC histological subtypes (e.g. keratinizing versus non-keratinizing). Given their distinct biological behaviors and prognostic implications, especially in the context of varying EBV associations, future international multicenter studies are warranted to validate these findings across diverse populations and subtypes. Furthermore, the current dataset lacked a comprehensive assessment of thyroid autoimmunity, specifically thyroid peroxidase (TPOAb) and thyroglobulin antibodies (TgAb), which precludes a deeper understanding of the autoimmune mechanisms linking thyroid function to therapeutic efficacy. Finally, the lack of central laboratory TSH standardization represents another limitation.

To address these gaps, future investigations should prioritize large-scale, prospective validation cohorts incorporating detailed thyroid panels. Concurrently, translational research is warranted to elucidate the biological underpinnings of this association, particularly by characterizing immune cell infiltration and tumor microenvironment profiles in patients with varying basal TSH phenotypes. It also remains to be determined whether the prognostic value of basal TSH is exclusive to KL-A167 or extrapolatable to other checkpoint inhibitors, such as anti-PD-1 agents. Given the distinct toxicity profiles, particularly the higher incidence of thyroid dysfunction associated with PD-1 inhibition, comparative studies are necessary to discern potential differences in clinical utility.

From a clinical perspective, the variations in monitoring practices highlighted in our study underscore the urgent need for standardized protocols across institutions to ensure data consistency. Ultimately, future research should assess the feasibility of utilizing basal TSH to tailor therapeutic regimens, potentially guiding decisions regarding treatment duration or the integration of combination strategies for patients exhibiting favorable baseline immunological features.

## Conclusion

Our study demonstrates that higher basal TSH levels are associated with improved PFS and OS in patients with recurrent/metastatic NPC receiving KL-A167. This finding suggests that basal thyroid function could be a useful prognostic biomarker for KL-A167 therapy response in this patient population. While the mechanisms underlying this association require further investigation, our findings may have clinical implications, including the potential to inform treatment assessment and improve risk stratification for this specific therapy. Future research should aim to confirm these findings in larger, prospective studies and explore the mechanisms linking basal thyroid function to ICI efficacy. In addition, the development of standardized protocols for monitoring thyroid function across different centers is essential for ensuring the consistency and reliability of such assessments.

In the context of the ongoing advancements in immunotherapy for NPC, our study provides new insights that could help in optimizing treatment strategies and improving patient outcomes.

## Supplementary materials



## Declaration of interest

The authors declare that there is no conflict of interest that could be perceived as prejudicing the impartiality of the work reported.

## Funding

The work was supported by the Noncommunicable Chronic Diseases National Science and Technology Major Project (2023ZD0503000 and 2023ZD0503004), the Regional Innovation and Development Joint Fund Key Project of the National Natural Science Foundation of China (U24A20735), the National Natural Sciences Foundation of China (82473434), the Fundamental and Interdisciplinary Disciplines Breakthrough Plan of the Ministry of Education of China, the Institutional Joint Innovation Fund from Sichuan University and Nuclear Power Institute of China (1-KJ-FWHT-WU-20240150), Sichuan Science and Technology Program (2025YFHZ0087), Science and Technology Project of Sichuan Provincial Health Commission (Clinical Research Special Project JH2023082), the International Science and Technology Cooperation Program of Chengdu Science and Technology Bureau (2024-YF06-00011-HZ and 2022-GH03-00004-HZ), the Health Research Project of Chengdu Eastern New Area Management Committee (202304), 1.3.5 project for disciplines of excellence from West China Hospital of Sichuan University (ZYYC23006), Clinical Research Incubation Project of West China Hospital (23HXFH001), Yunnan Province Key Laboratory of Precision Diagnosis and Treatment for Thoracic Diseases (202449CE340026), and the Ministry of Education University-Industry Collaborative Education Program (230720523707281). The funders had no role in study design, data collection and analysis, decision to publish, or preparation of the manuscript.

## Author contribution statement

K C, H F, and S X are co-first authors. They participated in the study design, data collection, and manuscript preparation, contributing equally to the final research output. All authors made a significant contribution to the work reported, whether that is in the conception, study design, execution, acquisition of data, analysis and interpretation, or in all these areas; took part in drafting, revising or critically reviewing the article; gave final approval of the version to be published; have agreed on the journal to which the article has been submitted; and agree to be accountable for all aspects of the work.

## Ethics

This study is registered with ClinicalTrials.gov (NCT03848286). All study procedures complied with the ethical standards outlined in the Declaration of Helsinki and the Good Clinical Practice (GCP) guidelines issued by the International Council for Harmonisation of Technical Requirements for Pharmaceuticals for Human Use (ICH).

## Data sharing statement

The data that support the findings of this study are available from Sichuan Kelun-Biotech Biopharmaceutical Co., Ltd (Chengdu, China), but restrictions apply to the availability of these data, which were used under license for the current study, and so are not publicly available. Data are, however, available from the authors upon reasonable request and with permission of Sichuan Kelun-Biotech Biopharmaceutical Co., Ltd (Chengdu, China).
