# Supplementary figures and images for: Association of basal thyroid function with clinical outcomes in patients with recurrent or metastatic nasopharyngeal carcinoma treated with PD-L1 inhibitor KL-A167: a multicenter post hoc analysis
Source: Endocr Connect. 2026 Apr 28;15(4):e260083. doi: 10.1530/EC-26-0083 (PMC13150324; doi:10.1530/EC-26-0083)

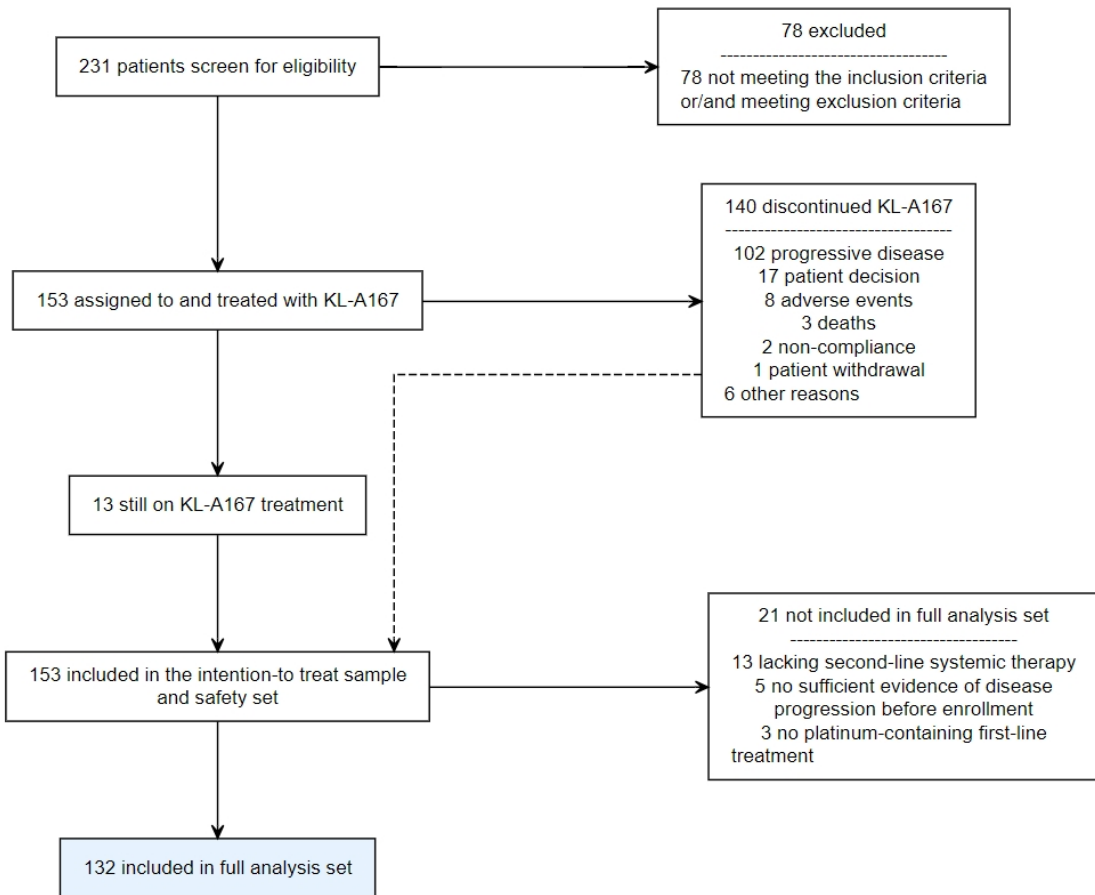

Supplement: Supplementary file 1 [file supplementary_materials.pdf]
